# ﻿A new freshwater species of *Pinnularia* (Bacillariophyta) from Hunan Province, China

**DOI:** 10.3897/phytokeys.237.116946

**Published:** 2024-01-23

**Authors:** San-Mei Xu, Bing Liu, Patrick Rioual, Man-Qi Yi, Yi-Dan Ma

**Affiliations:** 1 College of Biology and Environmental Sciences, Jishou University, Jishou, China Jishou University Jishou China; 2 School of Arts and Science, Huaihua Normal College, Huaihua, China School of Arts and Science, Huaihua Normal College Huaihua China; 3 Key Laboratory of Cenozoic Geology and Environment, Institute of Geology and Geophysics, Chinese Academy of Sciences, P.O. box 9825, Beijing 100029, China Institute of Geology and Geophysics, Chinese Academy of Sciences Beijing China; 4 CAS Center for Excellence in Life and Paleoenvironment, Beijing 100044, China CAS Center for Excellence in Life and Paleoenvironment Beijing China

**Keywords:** Central area, divergent striae, Huping Mountain, *Pinnulariahupingensis*, valvocopula

## Abstract

This study describes a new species of *Pinnularia*, *P.hupingensis***sp. nov.**, on the basis of light and scanning electron microscope images. *Pinnulariahupingensis***sp. nov.** is characterised by its linear valve outline, extremely divergent striae, and very large hexagonal central area occupying ca. 1/5–1/8 of the valve length. The primary and secondary sides of the valve and the internal proximal raphe fissures are discussed. The new species is compared to similar taxa of the genus *Pinnularia*.

## ﻿Introduction

The genus *Pinnularia* belongs to the artificial category of symmetric biraphid diatoms and is characterised by two girdle-appressed plate-like chloroplasts and alveolate striae (Round et al. 1990). According to the AlgaeBase website, there are 880 accepted species names, 501 accepted varieties and 127 accepted formae in the genus at present (Guiry and Guiry 2023). Hence *Pinnularia* is a very large and speciose genus, with numerous species described from Chinese material (e.g. Zhang et al. (2016); Liu et al. (2018a); Kociolek et al. (2020); Deng et al. (2021); Zhang et al. (2022)). The monograph of Krammer (2000), which provides the descriptions and illustrations of many *Pinnularia* taxa, still represents a very valuable source for taxon identification, although numerous other references are also needed since many new *Pinnularia* species have been described in the 21^st^ century. The different valve sizes, outlines, the arrangements and densities of the striae on the valve surface, the central areas, the shapes of the distal raphe fissures and the ornaments on the valve surface are the most useful characters to discriminate the taxa within the genus *Pinnularia* (e.g. Round et al. (1990); Krammer (2000)).

**Figure 1.**
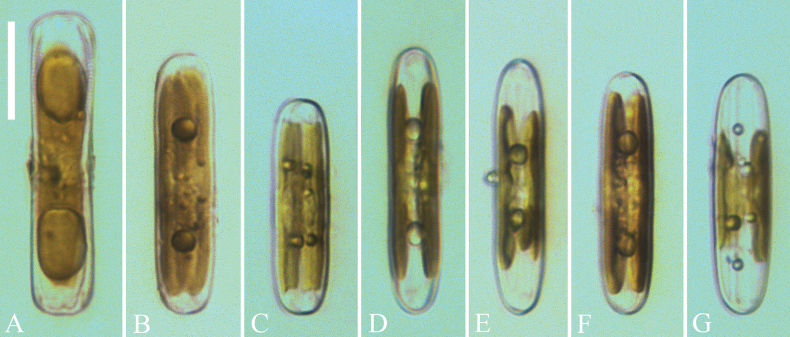
*Pinnulariahupingensis* sp. nov., LM**A–C** three living cells in girdle view, note that the girdle-appressed chloroplast spreads along the apical plane **D–G** four living cells in valve view, note the two plate-like, girdle-appressed chloroplasts. Scale bar: 20 μm.

**Figure 2.**
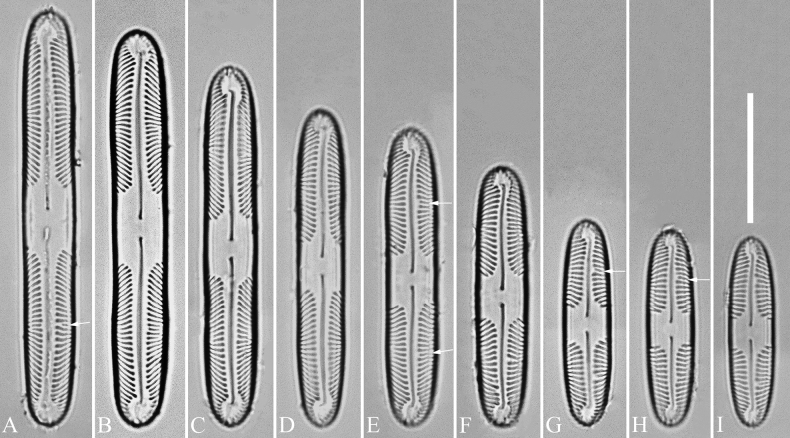
*Pinnulariahupingensis* sp. nov., LM**A–I** nine valves exhibiting a size diminution series, note the Voigt faults present in some of the valves (arrows on **A, E, G, H**) **B** micrograph of the holotype specimen. Scale bar: 20 μm.

**Figure 3.**
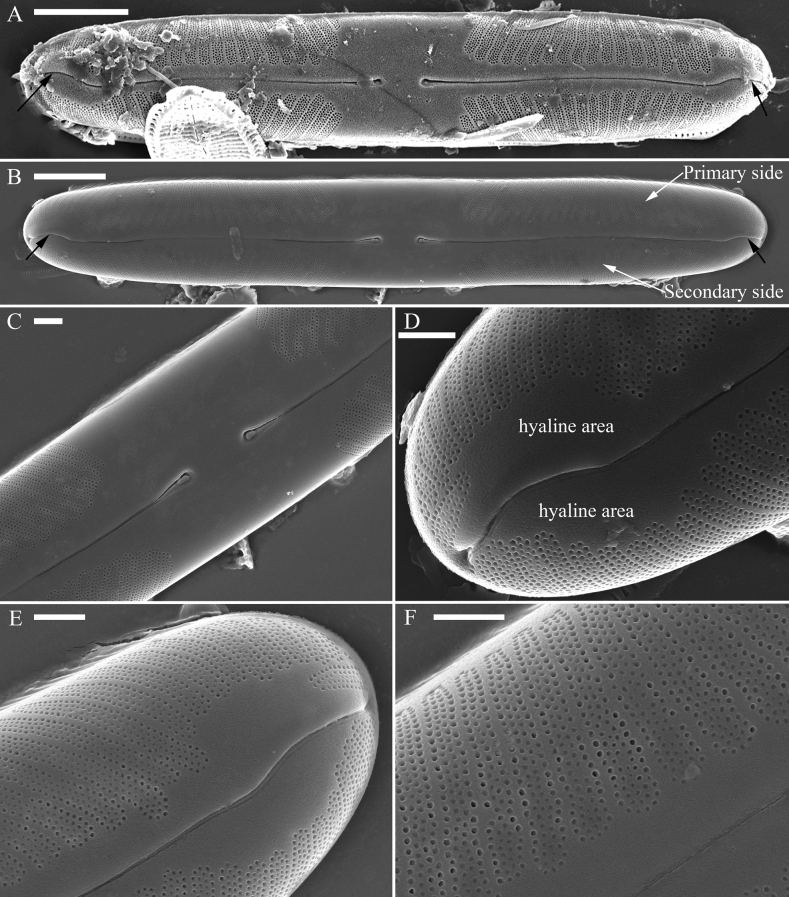
*Pinnulariahupingensis* sp. nov., SEM, valve external view **A, B** two complete valves, note the primary and secondary sides and the curved distal raphe fissures (black arrows) **C** middle part, details from **B** showing the large, hexagonal central area, slightly expanded proximal raphe endings bent in the same direction towards the primary side **D, E** apices, details from **B** showing the curved distal raphe fissures and apical hyaline areas **F** detail of the striae, note each stria comprises 3–5 rows of small round poroids. Scale bars: 5 μm (**A, B**); 1 μm (**C–F**).

**Figure 4.**
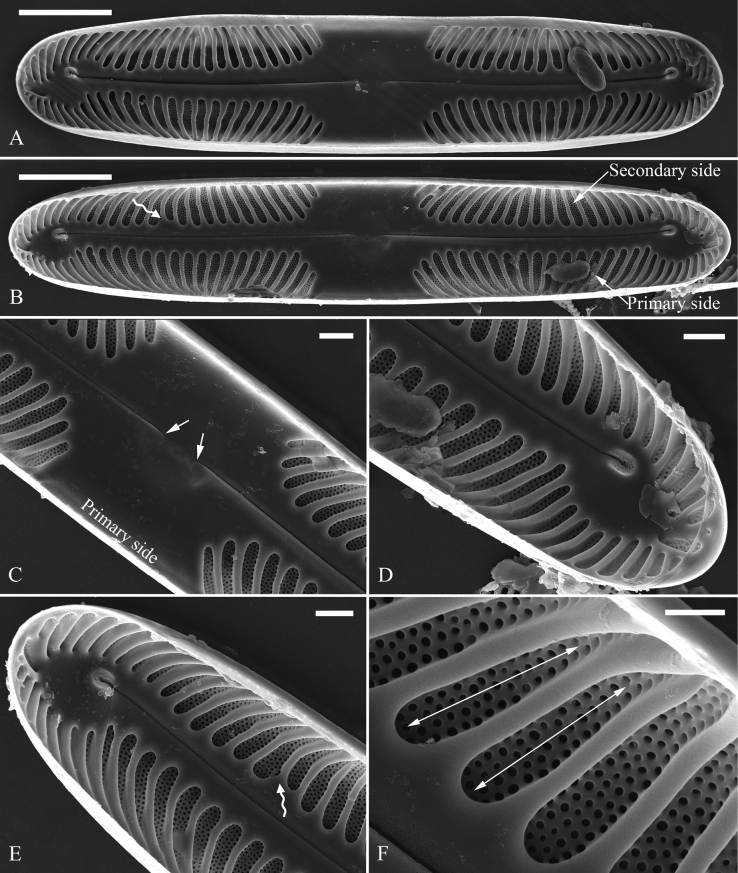
*Pinnulariahupingensis* sp. nov., SEM, valve internal view **A, B** two complete valves, note the primary and secondary sides **C** middle part, details from **B**, note the large, hexagonal central area and the internal proximal raphe fissures both deflecting towards the primary side (two arrows) **D, E** apices, details from **B**, note each internal distal raphe fissure running into a small, knob-like helictoglossa, the hyaline areas and the Voigt fault (**B, E**, wavy arrow respectively) **F** internal detail of the chambers, note the large transapical elongate apertures (two double-headed arrows). Scale bars: 5 μm (**A, B**); 1 μm (**C–F**).

**Figure 5.**
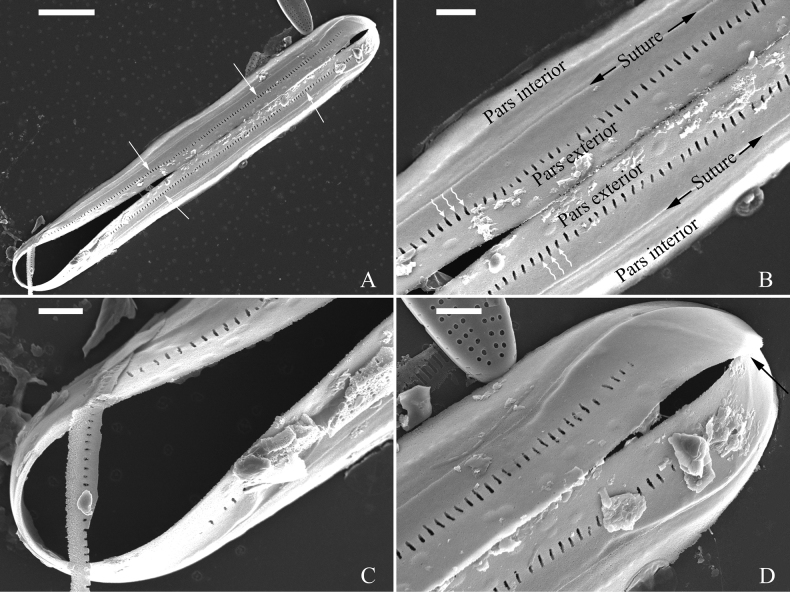
*Pinnulariahupingensis* sp. nov., SEM**A** one complete valvocopula, note a row of elongate poroids are produced in the pars exterior (four arrows) **B** middle part details from **A**, note the pars interior, suture, pars exterior and the elongate poroids (wavy arrows) **C** one apical detail from **A**, note the valvocopula is closed at this apex **D** the other apical detail from **A**, note the valvocopula is open at this apex (black arrow). Scale bars: 5 μm (**A**); 1 μm (**B–D**).

There are two main types of stria arrangements on the valve surfaces of *Pinnularia* taxa. In one type, the striae are radiate throughout the valve surface, such as in *P.hustedtii* F. Meister (Williams et al. 2022). In the other type, the striae are divergent, i.e. the striae are radiate only in the middle of the valve and abruptly become strongly convergent near the two apices, such as in *P.superdivergentissima* H. Germain & Chaumont (Chaumont and Germain 1976; Krammer 2000). If the central area in *Pinnularia* exists, it can be hyaline without any ornaments, such as in *P.lacustrigibba* Poulíčková, D.G. Mann & Kollár (Poulíčková et al. 2018) and *P.qinghainensis* Bing Liu & S. Blanco (Deng et al. 2021) or it may have lunate markings on each side of the central nodule, such as in *P.brandelii* Cleve (Krammer 2000, plate 97, figs 7–12) and *P.stomatophora* (Grunow) Cleve (Krammer 2000, plate 100, figs 1–8). Interestingly, in the closely-related genus *Caloneis*, the lunate markings are more common. Both *Caloneislewissi* Patrick and *C.schumanniana* (Grunow) Cleve have lunate markings on each side of the central nodule (Stancheva et al. 2009). Levkov and Williams (2014) observed *Caloneis* species from the ancient lakes Ohrid and Prespa and described eighty new species, all of them with lunate markings. The lunate markings seen under the light microscope (LM) are actually lunate depressions as seen under the scanning electron microscope (SEM) (e.g. Krammer (2000), plate 98, figs 7, 8; Stancheva et al. (2009), fig. 39). The stria arrangement and the central area are two important characteristics for the new species described in this paper as they can be used for its distinction from similar species.

Hunan Province is situated in south-central China. Most of the streams/rivers in Hunan are tributaries of four major river systems: the Xiang, Zi, Yuan and Li Rivers. These four major rivers run from the south of Hunan to its north and terminate in Dongting Lake, the largest lake in Hunan that discharges into the Yangtze River (the longest river in China). The diatom flora of Hunan remains underexplored, although Liu and his collaborators have described many new diatom species from Hunan in recent years (e.g. Liu et al. (2018b, 2019, 2020, 2021, 2022); Liu (2023)). This paper further contributes to the investigation of the diatom flora of Hunan by providing the description of a new *Pinnularia* species collected from a river from this Province.

## ﻿Materials and methods

The diatom samples of this study were collected from the Xie River which originates in the Huping Mountain National Nature Reserve (29°50'–30°09'N, 110°29'–110°59'E, 230 m a.s.l.). The Huping Mountain National Nature Reserve is located in the northwest of Shimen County, Hunan Province and is bordered by the Wufeng and Hefeng Counties of Hubei Province. This reserve was approved by the Chinese government as a national nature reserve in 1994 and it has a total area of 66.6 km^2^ and a core area of about 54.5 km^2^ (Tian et al. 2019). The Xie River is a headwater tributary of the Li River which is one of the four large rivers in Hunan Province. Epilithic diatom samples were collected on 14 March 2021. The method of collecting the diatom samples is the same as in Yuan et al. (2023) and consists of sampling numerous submerged stones showing yellow-brown surfaces that indicate the presence of diatoms. Each stone was placed on a plastic plate and its surface was brushed using a toothbrush, with the brushed-off diatom samples being washed onto the plate. The diatom samples were transferred into two 100 ml sampling bottles. One bottle was fixed with 70% ethanol and the other was left unfixed. At the time of sample collection, temperature, pH and conductivity were measured in situ with a portable multimeter (HQ40D, HACH Company).

The laboratory methods are also the same as in Liu (2023) and consist as follows: “The collected diatom samples to which 70% alcohol was not added were used to observe the living cells. A total of 100 μl diatom samples were transferred into a round chamber (diameter 14 mm, depth 0.35 mm) located in the middle of a custom-made slide by using a pipette, then examined using a Leica DM3000 light microscope (LM), equipped with a Leica MC190 HD digital camera. The collected diatom samples to which 70% alcohol was added were processed (cleaned) for microscopic examination with 10% hydrochloric acid (HCl) and 30% hydrogen peroxide (H_2_O_2_). Permanent slides were prepared using Naphrax mountant and examined using the same light microscope as above. Slides are deposited in the Herbarium of Jishou University, Hunan, People’s Republic of China (JIU) (Herbarium acronyms follow Index Herbarium http://sweetgum.nybg.org/science/ih/). Samples were also examined using scanning electron microscopy (SEM). Several drops of the cleaned diatom material were air-dried on to glass coverslips. The coverslips were attached to aluminium stubs using double-sided conductive carbon strips and sputter-coated with platinum (Cressington Sputter Coater 108auto, Ted Pella, Inc.). Samples were examined and visualised using a field emission scanning electron microscopy (FESEM) Sigma HD (Carl Zeiss Microscopy) available at Huaihua University, China”. The terminology in the diatom descriptions and in the discussion mainly follows Round et al. (1990) and Krammer (2000).

## ﻿Results

### ﻿ Pinnularia hupingensis

Taxon classificationPlantaeNaviculalesPinnulariaceae

Bing Liu & Rioual sp. nov.

DBA7073E-78DB-5FB8-B8CC-E8962E5C6CA1

Figs 1 , 2 , 3 , 4 , 5

#### Holotype.

Slide DIA202316, specimen circled on the slide, illustrated here as Fig. 2B, deposited in the Herbarium of Jishou University (JIU), China. Registration: http://phycobank.org/104258.

#### Type locality.

China. Hunan Province, Shimen County, Huping Town, a sampling site (29°57'6"N, 110°45'37"E, 230 m a.s.l.) in a riffle of the Xie River, collected by Bing Liu, 14 March 2021.

#### Description.

***LM*** (Figs 1, 2). Living cells rectangular in girdle view (Fig. 1A–C), linear with rounded apices in valve view (Fig. 1D–G). Two girdle-appressed, plate-like chloroplasts per cell (Fig. 1D–G). Valves linear with weakly undulate valve margins and broadly rounded apices. Valve dimensions (n = 28): length 28–65 μm, width 6.3–8.4 μm. Axial area narrow, raphe slightly undulate, filiform. Central pores small and bent towards the primary side and distal raphe fissures hooked. Central area very large (ca. 4.5–11.2 μm long), hexagonal, reaching both margins, occupying ca. 1/5–1/8 of the valve length. Striae extremely divergent, strongly radiate in the valve centre, becoming strongly convergent halfway to the apices. Striae 13–14 in 10 μm measured in the middle part of the valve near the central area. Voigt faults sometimes present on the secondary side (Fig. 2A, E, G, H, arrows). Apical hyaline areas well-developed.

***SEM*** (Figs 3–5). Valves linear with broadly rounded apices and flat surface, curving smoothly into relatively deep mantles (Fig. 3A, B). The valve primary and secondary sides can be recognised by the presence of Voigt faults on the secondary side (Fig. 3A, B). Central area very large, with two central pores both bent towards the primary side (Fig. 3A–C). Internally, proximal raphe endings interrupted by the central nodule, both turning towards the primary side (Fig. 4A–C). The external distal raphe fissures curved (Fig. 3A, B, D, E) while the internal distal raphe fissures run into a small, knob-like helictoglossa (Fig. 4D, E). Apical hyaline areas present (Figs 3D, E, 4D, E). Each alveolate stria comprises 3–5 rows of small rounded poroids externally (Fig. 3C–F) and internally, openings consist of elongate apertures (Fig. 4F, two double-headed arrows). Valvocopula has the same outline as that of the valve and is composed of pars interior, suture and pars exterior (Fig. 5A, B). Pars exterior ornamented by a row of elongate poroids (Fig. 5A, white arrows, Fig. 5B, white wavy arrows). Valvocopula closed at one apex and open at the other (Fig. 5C, D).

#### Etymology.

The specific epithet *hupingensis* refers to Huping Town where the species was found.

#### Distribution and ecology.

Known only from the type locality in which it is a common species with ca. 4% relative abundance. The samples that included this species were scraped off surface of stones collected in the Xie River. Hence, this is a benthic, epilithic species. The associated taxa include *Naviculareinhardtii* Grunow, *N.radiosa* Kützing, *Ulnariahupingensis* Bing Liu, *U.xieriverensis* Bing Liu and many unidentified *Cymbella* spp., *Fragilaria* spp., *Gomphonema* spp., amongst others. The following environmental parameters were measured in the field with three replications: Conductivity = 236.3 ± 1.2 μS cm^-1^; pH = 8.49 ± 0.02; Water temperature = 13.6 ± 0.1 °C.

## ﻿Discussion

*Pinnulariahupingensis* sp. nov. is characterised by its linear valve outline, extremely divergent striae and very large, hexagonal central area occupying ca. 1/5–1/8 of the valve length. The characteristics of *P.hupingensis* are summarised in Table 1 and compared to those of similar taxa. At first glance, *P.hupingensis* is similar to *P.brandelii*, but the latter has a lunate marking on either side of the central nodule (see Krammer (2000), plate 97, figs 7–12), whereas *P.hupingensis* does not have such lunate markings. Interestingly, Kezlya et al. (2022) noted that morphological distinctions, such as the surface markings in the central area, are consistent with phylogenic clades defined by genetic markers. The absence of such markings in *P.hupingensis*, therefore, excludes the possibility of this taxon being conspecific with *P.brandelii*. *Pinnulariabrebissonii* and *P.krammeri* are more or less similar to *P.hupingensis*, but the former two species have different valve outlines and central areas (Table 1). The relative size of the central area, in particular, has been reported to be a stable character for the recognition of *Pinnularia* species for both small and large specimens (Kollár et al. 2019). *Pinnulariahupingensis* differs from *P.superdivergentissima* by having narrower valves (6.3–8.4 vs. 8–10 μm in width) and much higher stria density (13–14 vs. 7–9 in 10 μm). Furthermore, the ratio between the length of the central area and the length of the valve is ca. 1/5–1/8 in *P.hupingensis*, but ca. 1/9–1/10 in *P.superdivergentissima*.

**Table 1.** Comparisons between *Pinnulariahupingensis* sp. nov. and similar taxa. Information on distribution collated from Algaebase (Guiry and Guiry 2023).

Taxon	*P.hupingensis* sp. nov.	*P.brandelii*	*P.brebissonii*	*P.krammeri*	*P.superdivergentissima*	*P.* sp1
Valve outline	Linear with weakly-undulate valve margins and rounded apices	Linear with broadly-rounded or broadly-capitate apices	Linear-lanceolate to linear-elliptical with broadly-rounded to wedge-shaped apices	Linear to linear- elliptical with wedge-shaped apices	Linear with commonly parallel sides and broadly-rounded apices	Linear with parallel or weakly-undulated margins, non-protracted, broadly-rounded apices
Valve length (L) and width (W) (μm)	L: 28–64, W: 6.3–8.4	L: 51–92, W: 7–10	L: 28–60, W: 9–12	L: 26–45, W: 6.7–7.8	L: 45–80, W: 8–10	L: 25–35, W: 5.0–6.5
Striae in 10 μm	13–14	11–14	10–12	11–13	7–9	11–13
Central area	Hexagonal, large, occupying ca. 1/5–1/7 of the valve length	Moderately broad to broad fascia, 1/7–1/9 of the valve length	More or less broad fascia, rarely with short striae at the fascia edge	Central area variable in shape and size, fascia widening to the valve margin to rhombic	Broad fascia, occupying ca. 1/9–1/10 of the valve length	Large rhombic fascia, occupying ca. 1/3 to 1/4 of the valve length
Lunate markings on each side of central nodule	Absent	Present, elongate	Absent	Absent	Absent	Absent
Type locality	Hubei, China	Finland, fossil	France	Finland, modern	France, Atlantic coast	Livingston Island, Antarctic Region
Distribution	Type locality	Arctic, Europe, N. America, Middle East	Cosmopolitan	Eurasia, N. America, Arctic, Antarctic	France, Britain, Ireland	Type locality
Reference	This study	Krammer (2000)	Krammer (2000)	Krammer (2000)	Krammer (2000)	Zidarova et al. (2012)

The most similar taxon to *P.hupingensis* is *Pinnularia* sp1, a taxon morphologically similar to *P.krammeri* illustrated in Zidarova et al. (2012, p.17, figs 44–47). *Pinnulariahupingensis* and *P.* sp1 both have linear valve outline, weakly undulate valve margins, divergent striae, large central area and overlapping valve dimensions and stria density (Table 1). Zidarova et al. (2012) did not provide a formal description and stated: “A few valves could not be identified but have been given a provisional identification as *Pinnularia* sp1 until further observations can be made”. Morphologically, *P.hupingensis* is very similar to *P.* sp1 of Zidarova et al. (2012) and it cannot be excluded that the two taxa may be conspecific.

During the ontogeny of raphid diatoms, the valve side formed from the initial branching of the raphe sternum is termed the primary side and its opposite side is termed the secondary side. The secondary side may include the Voigt faults (or discontinuities) that mark the point of fusion of the sternum during the ontogeny of the valve (Round et al. 1990). Most authors do not discuss the primary and secondary sides of the *Pinnularia* taxa they described (e.g. Krammer (2000)) including in recent publications (Liu et al. 2018b; Moreno et al. 2020; Deng et al. 2021; Kulikovskiy et al. 2023), although there are a few exceptions, such as Zidarova et al. (2012) and Poulíčková et al. (2018). In *P.hupingensis*, the Voigt faults could be seen on the majority of the valves we observed.

In *Pinnularia*, the internal proximal raphe fissures can be continuous or interrupted by the central nodule. The internal proximal raphe fissures are continuous in *Pinnularialacustrigibba* (Poulíčková et al. 2018) and *P.hustedtii* F. Meister (Williams et al. 2022), but interrupted by the central nodule in *P.hupingensis* as in *P.qinghainensis*. Clearly recognising these morphological structures is crucial to support the establishment of subgroups within the genus *Pinnularia* as discussed by Kulikovskiy et al. (2023).

## Supplementary Material

XML Treatment for Pinnularia hupingensis

## ﻿Additional information

### Author ORCIDs

San-Mei Xu https://orcid.org/0000-0003-1447-4752

Bing Liu https://orcid.org/0000-0002-8516-325X

Patrick Rioual https://orcid.org/0000-0001-9491-9197

Man-Qi Yi https://orcid.org/0000-0002-0503-0626

Yi-Dan Ma https://orcid.org/0009-0005-0636-4168

### Data availability

All of the data that support the findings of this study are available in the main text.
